# The Influence of Individualized Three-Dimensional Holographic Models on Patients’ Knowledge Qualified for Intervention in the Treatment of Peripheral Arterial Disease (PAD)

**DOI:** 10.3390/jcdd10110464

**Published:** 2023-11-15

**Authors:** Patryk Skórka, Michał Kargul, Diana Seemannová, Bartosz Gajek, Piotr Gutowski, Arkadiusz Kazimierczak, Paweł Rynio

**Affiliations:** Department of Vascular Surgery and Angiology, Pomeranian Medical University in Szczecin, Al. Powstańców Wielkopolskich 72, 70-111 Szczecin, Poland; p.skorka04@gmail.com (P.S.);

**Keywords:** augmented reality, mixed reality, HoloLens, peripheral artery disease, percutaneous transluminal angioplasty, patient education, hologram, informed consent, surgery preparation

## Abstract

We sought to determine the role of the patient-specific, three-dimensional (3D) holographic vascular model in patient medical knowledge and its influence on obtaining a more conscious informed consent process for percutaneous balloon angioplasty (PTA). Patients with peripheral arterial disease who had been scheduled for PTA were enrolled in the study. Information regarding the primary disease, planned procedure, and informed consent was recorded in typical fashion. Subsequently, the disease and procedure details were presented to the patient, showing the patients their individual model. A patient and medical supervisor equipped with mixed reality headsets could both simultaneously manipulate the hologram using gestures. The holographic 3D model had been created on a scale of 1:1 based on computed tomography scans. The patient’s knowledge was tested by the completion of a questionnaire before and after the interaction in a mixed reality environment. Seventy-nine patients manipulated arterial holograms in mixed reality head-mounted devices. Before the 3D holographic artery model interaction, the mean ± standard deviation score of the knowledge test was 2.95 ± 1.21 points. After the presentation, the score had increased to 4.39 ± 0.82, with a statistically significant difference (*p* = 0.0000) between the two scores. Using a Likert scale from 1 to 5, the patients had scored the use of the 3D holographic model at 3.90 points regarding its usefulness in comprehending their medical condition; at 4.04 points regarding the evaluation of the holograms as helpful in understanding the course of surgery; and rated the model at 1.99 points in reducing procedure-related stress. Using a nominal scale (know or don’t know), the patients had self-assessed their knowledge of the procedure before and after the 3D model presentation, with a score of 6.29 ± 2.01 and 8.39 ± 1.54, respectively. The study group tolerated the use of head-mounted devices. Only one patient had nausea and dizziness, while four patients experienced transient eye pain. The 3D holographic arterial model aided in the understanding of patients’ knowledge regarding the disease and procedure, making the informed consent process more conscious. The holograms improved the patient’s self-consciousness. Mixed reality headset-related complications were rare and within acceptable rates.

## 1. Introduction

Peripheral artery disease (PAD) is a chronic, mainly atherosclerotic disease of the peripheral vasculature resulting in limb-associated complications such as intermittent claudication, ischemic rest pain, ischemic ulcers, gangrene, and functional impairment [1]. PAD is the third leading cause of atherosclerotic morbidity, following coronary heart disease and stroke [2]. Pharmacotherapy, exercise therapy, and revascularization are the current treatment options for the limb symptoms of PAD, but each has its limitations [3]. A systematic review of 34 studies (22 from high-income countries and 12 from low- and middle-income countries) demonstrated that the prevalence of PAD was ≈5.8% at 50 to 54 years of age and ≈14.6% at 80 to 84 years of age in both men and women in high-income countries (HIC) and low-income or middle-income countries (LMIC) [4]. The awareness of symptoms, risk factors, and treatment options for PAD is low [5].

Therefore, it is essential that patients undergoing PTA are well informed about their medical condition, the procedure’s steps, possible complications, the expected results, and follow-up care. This can help them make an informed decision, improve their adherence to treatment, and enhance their satisfaction and quality of life.

There are significant barriers in communication between physicians and patients regarding PTA. One of the main challenges is the complexity and diversity of vascular anatomy and pathology, which can be difficult to explain and visualize using conventional methods, such as diagrams, models, or images.

Cognitive impairment of vascular etiology is the second most common cause of dementia and may be the predominant one in East Asia. Furthermore, alterations of the large and small cerebral vasculature, including those affecting the microcirculation of the subcortical white matter, are key contributors to the clinical expression of cognitive dysfunction caused by other pathologies, including Alzheimer’s disease [6]. As a result, a possible challenge in communication with patients is the heterogeneity of the patient population, which may include elderly people, people with cognitive impairments (such as age-related dementia or vascular dementia), or people with low health literacy or language barriers. These factors can limit the patients’ ability to understand and retain the information provided by the physicians.

To overcome these challenges, we propose to use a mixed reality (MR) headset to demonstrate to patients the holograms of their vascular system based on their computed tomography (CT) scans. There are different variants of blending reality with the digital world. Augmented reality (AR) uses digital elements such as the camera in a smartphone to preview live reality. Virtual reality (VR), which has been in use for several years and is used in many articles [7], is limited to total immersion and separation from the physical world. The user moves through the virtual environment and interacts with it in near real time.

MR is a technology that combines virtual and real elements in the same environment, creating an immersive and interactive experience. By using MR, we aim to enhance the patient’s understanding of their anatomy and pathology, as well as the steps and outcomes of the procedure. We also aim to verify the feasibility and acceptability of using MR in patients with different characteristics and needs.

In this paper, we present the design and implementation of the MR system for PTA education. We also report the results of a pilot study that evaluated the usability and usefulness of our system among patients scheduled for PTA in the treatment of PAD. To our knowledge, this is the first study to use MR for PTA education in a clinical setting.

## 2. Materials and Methods

### 2.1. Study Design and Setting

This was a pre–post intervention study that evaluated the effectiveness of MR education in patients undergoing PTA for the treatment of PAD. The study was conducted at the Department of Vascular Surgery of the Pomeranian Medical University in Szczecin, Poland, between November 2022 and January 2023.

### 2.2. Study Population and Sampling

The study population consisted of patients scheduled for lower limb PTA for PAD. The inclusion criteria were age 18 years or older, diagnosed chronic limb ischemia IIb F confirmed by CT scan, and able to give informed consent. The exclusion criteria were contraindication for PTA, severe visual or hearing impairment, severe cognitive impairment, or previous experience with MR technology.

A total of 80 patients met the inclusion criteria and agreed to participate in the study. The patients received MR education.

### 2.3. Three-Dimensional Hologram Preparation

For all patients who were included in the study, three-dimensional, personalized 1:1 scale models were created based on CT images taken before the procedure on various CT scanners. The creation of three-dimensional models covered the bones and arteries of the lower limbs with clearly marked stenosis and arterial obstructions, which are the cause of ischemia and the purpose of interventional treatment. Segmentation and modeling were performed in the open-source software 3DSlicer 5.3.0 [8]. The generated STL models were transferred to the Virtual Surgery Intelligence software 1.9. (Apoqlar, Hamburg, Germany).

### 2.4. Data Collection and Analysis

**Figure 1:**
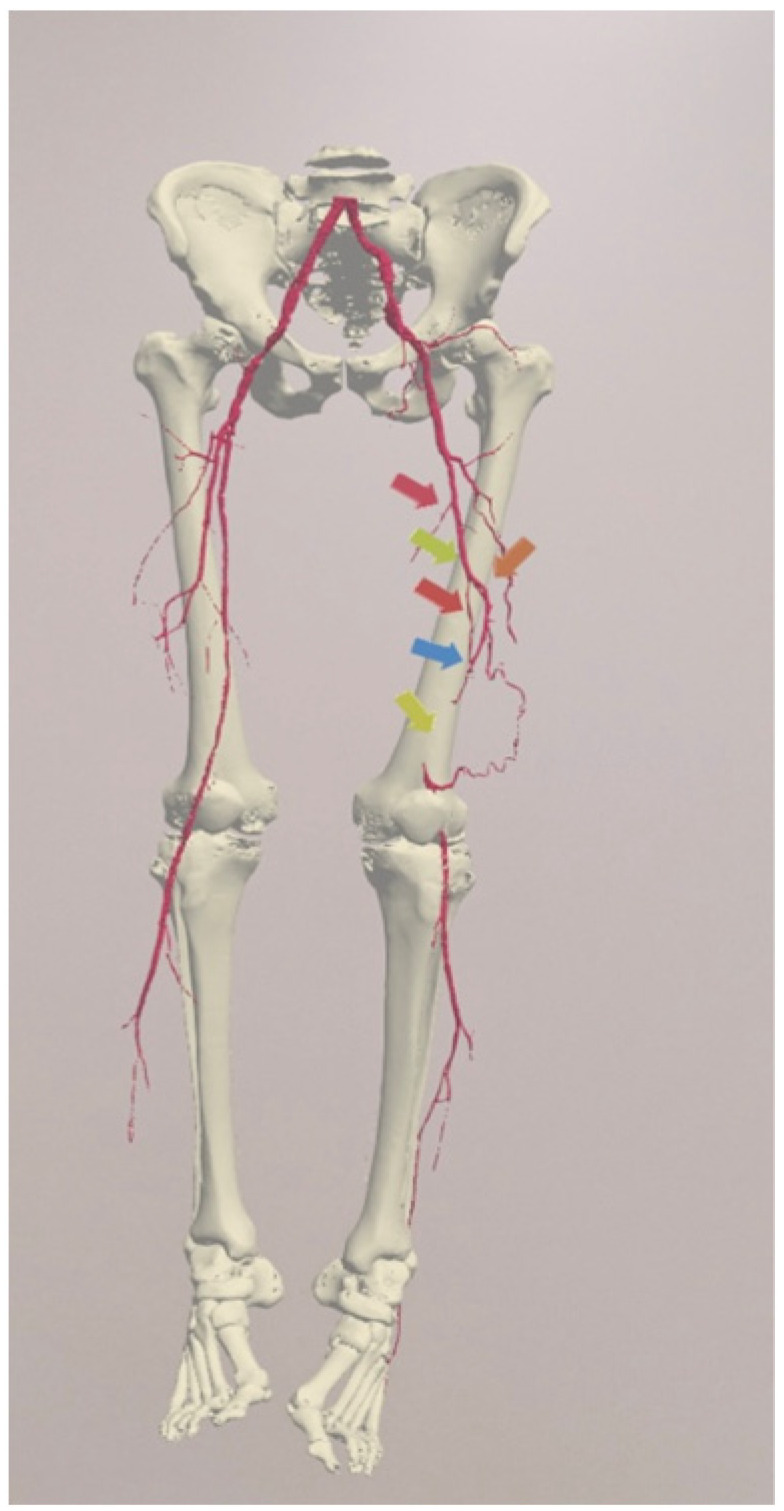
The hologram made available to the patient with the indication of the obstruction. Arrows indicate stenosis and obstruction of the patients arteries.

Data collection consisted of two phases: pre-education and post-education. During pre-education, before the MR education session, but after the conventional information session presented by a vascular surgeon, the patients completed a demographic questionnaire that collected information on their age, sex, education level, occupation, and comorbidities (Table S1). They also completed a specially designed questionnaire test to assess their knowledge about PAD and PTA (Figure 1).

After the education session, the patients completed the questionnaire test again to measure their knowledge gain. This included four additional questions that asked the patients to rate the usefulness of the holographic models in enhancing their understanding of their medical condition, the course of surgery, and reducing their stress, using a Likert scale from 1 (not at all) to 5 (very much). The tutor also asked the patients to self-assess their knowledge before and after the 3D model presentation.

Undesirable sensations and symptoms such as dizziness, headache, nausea, eye pain, imbalance, and anxiety were reported during the sessions.

### 2.5. Intervention

All patients received MR education using our system. The education session was delivered the day before the PTA procedure, in a separate room with no distractions. The education session lasted for about 15 min and was delivered by a trained researcher who followed a standardized script. The researcher did not provide any additional information or feedback to the patients.

The MR system consisted of two pairs of Microsoft HoloLens 2 glasses and a wireless router. The tutor shared a 3D model of the patient with the hologram sharing mode, and then the tutor indicated the obstruction to the patient by marking it and provided information about the procedure (Figure 1). Then, the patient independently tried to manipulate the hologram by performing activities such as: finding an occluded vessel, enlarging, scaling, and rotating. At the same time, the tutor assessed the patient’s ability to use the HoloLens glasses and the unpleasant feelings associated with it. After presenting the model to the patient, the tutor asked questions related to the procedure again, during which time he assessed how their awareness of the procedure had changed.

### 2.6. Statistical Analysis

The data analysis was performed using Statistica 13.3. Descriptive statistics were used to summarize the demographic characteristics and knowledge scores of the MR group. Continuous variables were presented as the mean and standard deviation and were checked for normality with the Shapiro–Wilk test. Paired *t*-tests and Wilcoxon tests were used to compare the pre-education and post-education knowledge scores within the MR group. The level of significance was set at *p* < 0.05.

## 3. Results

In total, 80 participants (mean age 68.55 ± 8.57) were included. Twenty-three were women (28.75%) and fifty-seven (66.25%) were men. In addition, 54 (67.5%) patients were educated to high school level, 14 (17.5%) to primary school level, and 9 (11.25%) to university level. All of the patients (80) were diagnosed with chronic limb ischemia IIb F.

**Figure 2:**
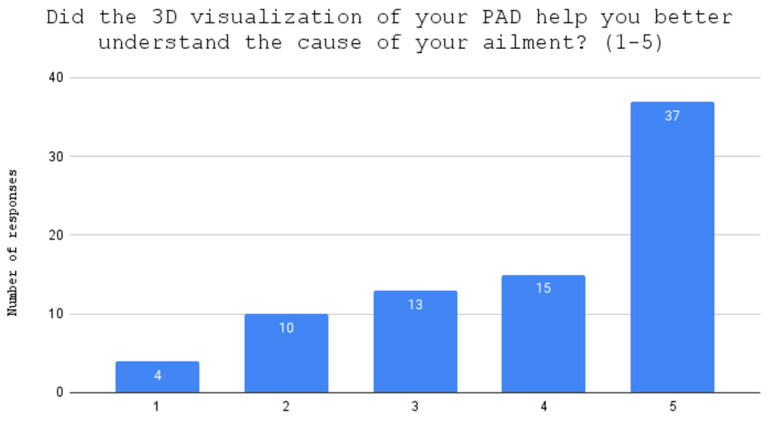
Evaluation of holograms as helpful in understanding the medical condition.

**Figure 3:**
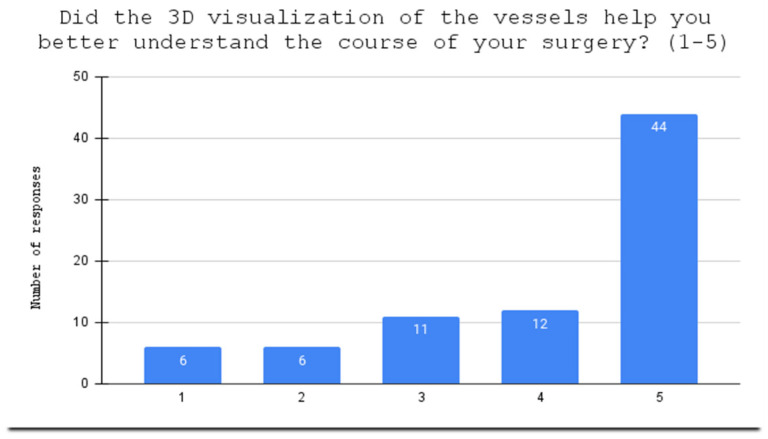
Evaluation of holograms as helpful in understanding the course of surgery.

**Figure 4:**
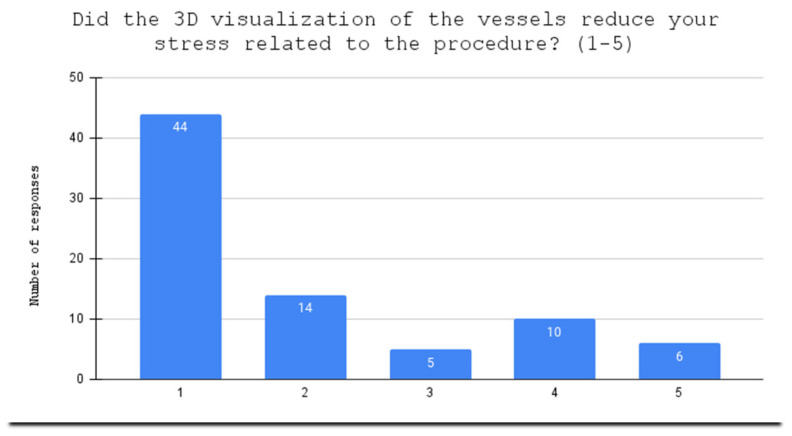
Evaluation of holograms as helpful in stress related to the procedure.

**Figure 5:**
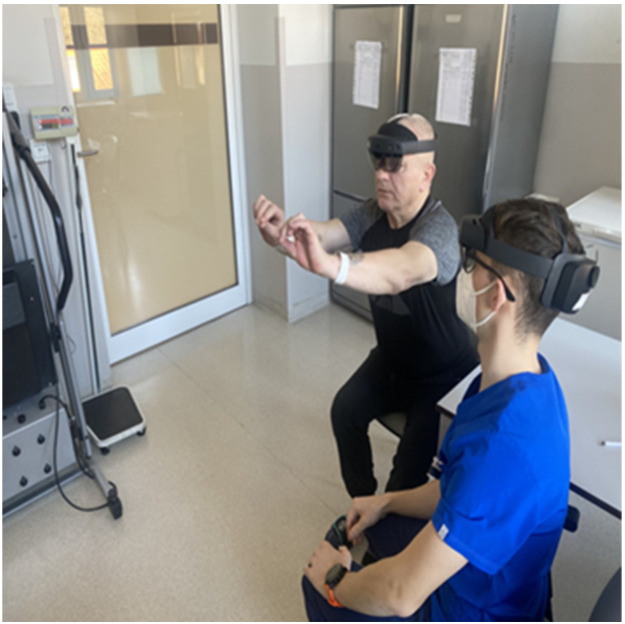
A patient manipulates the vascular hologram using the MR headset.

One patient, after answering part of the questionnaire, resigned from wearing the HoloLens2; therefore, seventy-nine patients manipulated arterial holograms in mixed reality head-mounted devices. Before the 3D holographic artery model interaction, the mean score of the knowledge test was 2.95. After the presentation, the score increased to 4.39, with a statistically significant difference (*p* = 0.0000) between the two scores. Using a Likert scale from 1 to 5, the patients scored the usefulness of holographic models at 3.90 points in the understanding of their medical condition (Figure 2); at 4.04 points in the evaluation of the holograms as helpful in understanding the course of surgery (Figure 3); and stress reduction was rated at 1.99 points (Figure 4). Using a nominal scale (know or don’t know) where patients could score a total of 0 to 10 points (0 being don’t know; 1 being know), patients self-assessed their knowledge before and after the presentation of the 3D model, scoring 6.29 and 8.39, respectively (Table S2). The results can be found in Table 1. Furthermore, all of our patients indicated the vessel on the scene while using the goggles (Figure 5), 65 patients had rotated the object, 48 patients had zoomed in on the vessels, and 42 had scaled. The outcomes are presented in Table 2. Sixty-two patients answered “YES” to the following question: “Should information about the procedure be provided with the use of HoloLens2 goggles?” Additionally, while using the HoloLens2 goggles, some patients reported the following symptoms: dizziness, headache, and eye pain. Almost all of our patients had no symptoms (Table 3). Nobody stopped using the goggles.

## 4. Discussion

The results of this study can be used to formulate and improve patient education initiatives that will reduce the risk of adverse cardiovascular and limb events [9]. We evaluated the effectiveness of using 3D vascular holograms in the education of patients with PAD who were scheduled for PTA. The patients had received standard education from a vascular surgeon before MR education, which resulted in medium self-confidence and satisfaction. MR education using our system was delivered after standard education, and it significantly improved the patients’ knowledge and awareness of their condition and procedure, as well as their self-confidence and preference for this method. MR education was feasible and acceptable for patients with different characteristics and needs, and it did not cause any major discomfort or adverse effects. Unpleasant feelings associated with using MR technology were rare and did not influence the education process in most patients.

Our study provides evidence that MR education can be a valuable tool for enhancing patient–surgeon communication, informed consent, and patient empowerment. By using MR education, we can help patients to visualize their own anatomy and pathology, as well as the steps and outcomes of the procedure, in a realistic and interactive way. This can increase their engagement, motivation, and confidence in their treatment decision and outcome.

Medical informed consent should be an exchange of ideas that buttresses the patient–physician relationship. The consent process should be the foundation of the fiduciary relationship between a patient and a physician [10].

Previous studies have shown that patient education can improve patient satisfaction, compliance, and quality of life, as well as reduce anxiety, decrease patient readmissions, and reduce costly errors. Improving communication and using technology to create more effective patient communication in healthcare will reduce errors and save lives [11].

Such findings are consistent with prior studies reporting an increase in the satisfaction of better-informed patients [12], based on a review by Alanazi et al. [13], which states that preoperative patient education reduces preoperative anxiety in patients scheduled for various surgical procedures.

In accord with another review [14], good doctor–patient communication has the potential to help regulate patients’ emotions, facilitate the comprehension of medical information, and allow for better identification of patients’ needs. Patient education methods should be tailored to the individual’s needs. Therefore, various educational concepts are used in patient education such as: written materials, videotapes, audiotapes, verbal instruction and demonstration, and mixed reality methods. As reported in another review [15], when using written educational methods, a wide range of factors should be considered to maximize their effectiveness. This consists of content, language, organization, layout and topography, illustration, learning, and motivation. The next strategy for education is videotapes; Mansell’s group has created videos for patients before lung transplantation. They proved that nearly half of the participants (8/17; 47%) explicitly indicated how much they appreciated genuine accounts [16]. The learning through listening method is currently very popular in patient education. Based on the review below, it can be concluded that podcasts on medical education reach a wide audience. Additionally, podcasts hold the potential to be powerful tools for disseminating innovations and evidence [17].

However, conventional methods of patient education, such as diagrams, models, or images, may not be sufficient to convey the complexity and diversity of vascular anatomy and pathology, or to address the heterogeneity of the patient population. MR education can overcome these limitations by providing a personalized, immersive, and interactive experience. In addition, based on a review by Friedman et al., [18] computer teaching strategies are effective when patients are given information specific to their own situation rather than general information. The use of VR glasses in patients also demonstrated a satisfactory effect. The application of VR in patient education is a promising technology. The widespread use of virtual reality allows it to be used in the doctor–patient relationship [19].

In addition, the feature of the HoloLens glasses, compared with other alternative media types, is that the HoloLens allows for full interaction between two or more users and for study participants to manipulate the image. However, each study participant must be trained in the use of the glasses and have the glasses, as determined by Hilt’s study [20].

Mixed reality has wide applications in vascular surgery. Technological advances have changed the face of vascular intervention over the last few decades. Augmented reality with holographic imaging of CTA data is helpful during EVAR and has the potential to improve perioperative outcomes [21]. As reported in the review by Eves, AR can increase accuracy and reduce procedure time while reducing radiation exposure and contrast dose [22]. Furthermore, the use of 3D image fusion can be applied to PTA iliac artery obstruction. As reported in the case report by Goudeketting, this method affects the accuracy of the image fusion technique [23].

Hatzl’s group [24] used MR to educate patients with abdominal aortic aneurysms. MagicLeap 1 was used; both the tutor and the patient observed the same virtual model, but only the surgeon could manipulate the model. In our study, both the tutor and the patient could manipulate the virtual model. The average patient satisfaction score in the MR group was as high as in our work. In comparison, work using 3D-printed models in kidney cancer patients has similarly confirmed the effectiveness of using virtual reality in patient education, as has our work [25]. In contrast to our work, a scale of 1–10 was used to assess the usefulness of the model, which enabled a more accurate assessment of the usefulness of the 3D models used. In our work, we have reported a level of satisfaction in patients with the ability to have a 360-degree view of the model, and while this has also been confirmed in work using VR, the ability to manipulate the object in VR is limited [26]. Education supplemented with VR is an effective strategy for educating students. As reported in a survey conducted by Kolecki, the majority of students and faculty surveyed believed that this mode of education was superior to classical methods. In addition, the ability to share a screen between the tutor and the student allowed for more lasting learning [27]. In our study, it led to improvement and modernization of the process of informing patients about their disease. The majority of our patients were able to indicate the vessels on the hologram (75/79), rotate the object (65/79), zoom in on the vessels (48/79), and scale (42/79), but we cannot compare the results in this area with others because other studies have not assessed the patient’s ability to manipulate the object. The use of a three-dimensional (3D) holographic model helps to build a meaningful relationship with the patient, increase patient satisfaction, and reduce costly errors. This is proven by the satisfactory effects of using telemedicine. As reported by Kruse’s group, patients’ expectations were met when providers delivered healthcare via videoconference or any other telehealth method [28]. Patients were highly satisfied and found VR education tools to be useful as they enhanced understanding, improved communication with healthcare professionals, and increased compliance with treatment [29]. Poor communication with the patient leads to negative consequences, such as reduced patient satisfaction, medical errors, and unclear indications. Doctor–patient communication is perhaps the most important “non-specific” or placebo effect in medicine [30]. The use of HoloLens glasses allowed patients to understand the course of the procedure and the reason for hospitalization more effectively. Based on Sweller’s theory of cognitive load [31], it can be concluded that the presented 3D holograms, as an unconventional method, provide a low level of cognitive load, and therefore increase the effectiveness of patient education more than in conventional methods. The method used is like the method in [32], which points to the direction and visual tools such as HoloLens2 for patient education. The use of HoloLens2 glasses proves the growing use of virtual reality, as well as the growing benefits of it [33]. This is the first study to involve educating patients with vascular disease using holograms.

There were several limitations to this study that we acknowledge and plan to address in our future research. Firstly, this study did not have a control group that either did not receive any education or received a different type of education, such as video or verbal explanation. This limits our ability to compare the effectiveness of MR education with other methods and to isolate the effects of MR education from other factors, such as the researcher’s interaction or the patient’s expectations. This is a new, rapidly developing technology that the review by Tang in *The Canadian Medical Education Journal* reports is currently at an early stage and lacks evidence-based support for widespread implementation [34]. Therefore, we plan to conduct a randomized controlled trial with a larger sample size and a longer follow-up period to evaluate the impact of MR education on the patient’s adherence, outcome satisfaction, and information recall. We also plan to compare MR education with other modalities of education, such as video or verbal explanation, to determine the optimal method for different patient groups and scenarios. Secondly, we did not assess the patients’ cognitive function before or after MR education. This may affect their ability to understand and retain the information provided by the MR system. We plan to include a measure of cognitive function in our future studies to examine its influence on the effectiveness of MR education.

## 5. Conclusions

The 3D holographic arterial model aided in the understanding of patients’ knowledge regarding the disease and procedure, making the informed consent process more conscious. The holograms improved the patients’ self-consciousness. Mixed reality headset-related complications were rare and within acceptable rates. 

## Figures and Tables

**Table 1:** Results of the knowledge test.

	Number of Patients	Average (Points ± SD)
Patients’ self-assessment before	79	2.95 ± 1.21
Patients’ self-assessment after	79	4.39 ± 0.82
Knowledge test score before	80	6.29 ± 2.01
Knowledge test score after	80	8.39 ± 1.54
Quality of information provided (1–5)	79	4.64 ± 0.72

**Table 2:** Evaluation of patients HoloLens manipulation skills.

Was the Patient Able to Manipulate the Googles Independently?	Number of Patients	Percentage of People
Indicate vessels on the hologram	75	94.94
Rotate the object	65	82.28
Zoom in on the vessels	48	60.76
Scale	42	53.16

**Table 3:** Side effects.

Did Any of the Following Symptoms Occur?	Number of Patients	Percentage of People
Dizziness	1	1.27
Headache	1	1.27
Nausea	0	0.00
Eye pain	4	5.06
Balance impairment	0	0.00
Anxiety	0	0.00

## Data Availability

The data that support the findings of this study are available from the corresponding author upon reasonable request.

## Supplementary Materials

The following supporting information can be downloaded at: https://www.mdpi.com/article/10.3390/jcdd10110464/s1, Table S1. Patient Knowledge Questionnaire on PTA i PAD; In the first question we used the Likert scale (1–5), in the following questions we used the nominal scale (know or don’t know); Table S2. Demographic data and comorbidities of the patients.
