# Supplementary material for: The Influence of Individualized Three-Dimensional Holographic Models on Patients’ Knowledge Qualified for Intervention in the Treatment of Peripheral Arterial Disease (PAD)
Source: J Cardiovasc Dev Dis. 2023 Nov 15;10(11):464. doi: 10.3390/jcdd10110464 (PMC10671973; doi:10.3390/jcdd10110464)
Supplement: Supplementary file 1 [file jcdd-10-00464-s001.zip › jcdd-2674644-supplementary.pdf]

Supplementary Materials

Table S1. Demographic data and comorbidities of the patients

	Number of patients	Percentage of people
Age (years), mean ± SD	68.55 ± 8.57	
Female sex	23	28.75
Male sex	57	66.25
Education		
Primary school	14	17.5
High school	54	67.5
University degree	9	11.25
Comorbidities		
Hypertension	64	81.01
DM2	28	35.44
Asthma	8	10.13
Chronic obstructive pulmonary disease	5	6.33
PAD	79	100.0
Coronary artery disease	25	31.65
Atrial fibrillation	13	16.46
TIA	4	5.06
Chronic kidney disease	8	10.13
Dementia	3	3.80
Parkinson's disease	0	0.00
Other mental disorders	0	0.00
Surgery history		
CABG	12	15.19
PTA	33	41.77
PTCA	15	18.99
Smoking	6	7.5
Non or former smoker	74	92.5

Table S2. Patient Knowledge Questionnaire on PTA i PAD; In the first question we used the Likert scale (1-5), in the following questions we used the nominal scale (know or don't know).

Question	Before	After
What procedure will be performed?		
Which vessel is obstructed?		
What is a thrombosis?		
What are the symptoms of ischemia?		
What is the procedure of PTA?		

In which part of the body will the procedure be performed?
How is PTA performed?
What happens if ischemia is not treated?
Why is PTA done?
